# Mechanical Properties of Carbon-Fabric-Reinforced High-Strength Matrices

**DOI:** 10.3390/ma13163508

**Published:** 2020-08-09

**Authors:** Bekir Yılmaz Pekmezci, Ali Çopuroğlu

**Affiliations:** Istanbul Technical University, Faculty of Civil Engineering, Maslak, Istanbul 34469, Turkey; copuroglu16@itu.edu.tr

**Keywords:** flexural strength, tensile strength, high-strength concrete, cementitious composites, FRCM, TRC, fabric-reinforced cementitious composite, textile-reinforced concrete

## Abstract

Fabric-reinforced cementitious matrices (FRCM) are promising technologies that respond to today’s architectural approaches. However, due to their high strength and ductility, they are starting to be implemented in buildings as strengthening systems. In this experimental study, the amount of fiber along the load direction in high-strength cementitious matrices and the effects of the fiber orientation on FRCM mechanical properties were studied. A total of four different composites were produced with two fabrics and two matrices. Tensile and flexural tests were carried out on composites. Within the scope of microstructure studies, scanning electron microscope micrographs were obtained and analyzed, along with microtopography sections. The main result obtained from the study indicates that as the fiber area in the direction of the load increases, the load order carried in this direction increases. However, this increase does not have to be proportional to the fiber area used in the direction of the load. The fiber coating and coating matrix interface play important roles in a composite’s performance. The carbon fibers can be used more efficiently by using them along the load direction and the loads in the matrix can be transferred to the carbon fibers by creating a larger fiber–matrix interface area.

## 1. Introduction

Cementitious materials with high compressive strength, low tensile strength, and high toughness are generally strengthened with reinforcement. In existing applications, conventional concrete reinforcement steels are used as reinforcement. Reinforced concrete is a system that has been widely used all over the world for decades. Reinforced concrete has proved itself by showing success under many conditions. Accordingly, the calculation methods have improved considerably and engineers have a high level of experience and confidence in reinforced concrete calculation methods. However, reinforced concrete elements should be designed with certain dimensions to provide the desired performance [1,2,3]. In today’s architectural approach, structures are designed with the use of smaller cross-section elements. These elements are produced with small aggregate sizes. For this, high performance and small section reinforcements are needed [4]. These elements, which are generally produced with cement-based materials, have been reinforced with discrete fibers for a long time to provide the required high tensile strength, toughness, and ductility [1,2,3]. Discrete fibers used in the production of cementitious composites can be listed as acrylic, cotton, asbestos, glass, basalt, carbon, nylon, polyester, polyethylene, polypropylene, steel, and polyvinyl alcohol [5,6]. When fibers are classified according to their base, it is understood that they are polymer-, metal-, and mineral-based. The fibers show differences in their strength and elasticity moduli. While carbon and steel have the highest modulus of elasticity, polymer-based fibers have the lowest modulus of elasticity [3,4]. Despite the long history of fiber-reinforced concretes, fibers in fabric form have also started to be used for reinforcement thanks to the developing new textile technologies [7,8]. Fabrics allow easy production of cementitious composites and high mechanical adhesion [9]. While the discrete fibers are randomly distributed in the composite, the fibers are used more effectively, as the fibers are oriented in the desired and needed direction when used as fabric [10]. Moreover, when using fabrics, the path that cracks follow is changed, which increases the ductility [7].

Composites produced using the mentioned fabrics are called fabric-reinforced cementitious matrices (FRCMs) or textile-reinforced mortars (TRM). FRCM composites can be obtained using different fabrics and different matrices. The raw materials of fabrics are similar to those of fibers and can be glass, basalt, carbon, polypropylene, polyvinyl alcohol, sisal, and p-phenylene benzobis oxazole [11,12,13,14,15,16,17]. These fabrics can be woven, non-woven, or knitted. The yarn dimensions, distribution of filaments, penetration of the matrix through the fibers, and the bond at the interface affect the fracture behavior of a composite [3,18,19]. The matrices can be cement-, lime-, or geopolymer-based [20,21]. The properties of the raw materials used in production and the properties of the components of the composite vary. To ensure sufficient bonding when using dry fabric, penetration must occur by using the appropriate fiber type, and cement particles must pass through the filaments to form the fabric [22,23,24]. The compatibility of the fabric and the matrix and the properties of the coatings used at the fabric–matrix interface play important roles in the performance of the composite [15]. When the fibers are coated with polymer-based coatings, the bonding character depends on the compatibility of the matrix with these coatings and the properties of this coating [25,26]. Joints in the fibers in fabric also contribute to increased bonding [24].

Composites obtained using low-, normal-, and high-strength matrices are used in many fields. These composites are used in the production of some structural elements [10], permanent molds, cladding panels [27,28], and strengthening elements of existing structures [11,29]. It is known that FRCMs are used to reinforce pipes [30]. To obtain high bonding and high composite performance, high-strength matrices are used in addition to normal-strength matrices. Composites produced with medium- and high-strength cementitious matrices are generally used for exterior cladding of buildings or strengthening of masonry and reinforced concrete structures [10,11,27,28,29,31]. Sandwich panels produced using FRCM can meet the high mechanical performance requirements expected from them. Various core materials have been used for these types of sandwich panels and analytical models have been developed to predict their mechanical behavior [32,33,34,35]. FRCM composites significantly increase the mechanical properties of reinforced concrete elements [25,36,37] and masonry [38,39,40]. The high temperature resistance of FRCMs is one of their most important advantages [41].

In addition to high-strength concretes, ultra-high-performance matrices are also used in FRCM production in practice. In ultra-high-performance concretes, discrete fibers are often used to obtain high ductility and toughness levels in concretes [42]. The bond performance of ultra-high-performance concrete (UHPC) and its compatibility with conventional reinforcement are well known [43,44,45]. However, the compatibility of fabrics with UHPC caught the attention of a few researchers, who have started to work on this subject [46]. Ultra-high-performance concrete is a class of advanced cementitious materials with greater strength, tensile ductility, and durability properties when compared to conventional or even high-performance concrete, according to American Concrete Institute Committee 239 Recommendation (ACI239-R18) [47]. The advanced properties of ultra-high-performance mortars emerge as a result of their designs being different from traditional concrete. Here, very fine binders, supplementary cementitious materials, and filler materials have significant effects [48,49,50].

Cementitious matrices with fabric reinforcement are generally characterized by tensile and flexural test results. Although flexural tests are sufficient for composites used for exterior cladding, it is recommended to do tensile tests on composites used as strengthening systems. Tensile experiments are usually carried out according to two regulations. One of these documents is International Union of Laboratories and Experts in Construction Materials, Systems and Structures Technical Committee 232 Recommendation (RILEM 232) [51], while the other is International Code Council-Evaluation Service—Acceptance Criteria (434 ICC-ES AC434) [52] document, which is cited in American Concrete Institute Committee 549 Recommendation (ACI 549.4R-20) [53].

In this experimental study, the carbon fabric produced by using carbon fibers in different proportions in two different directions was used in high-strength matrices. Fabric-reinforced cementitious matrix (FRCM) composites were produced by employing these fabrics in two different types of high-strength cementitious matrices. The properties of the produced FRCM composites were investigated. Tensile and flexural tests were performed on composites produced with two different matrices and two different carbon fabrics. In addition, microstructural investigations were performed with a scanning electron microscope (SEM).

Energy is used in the production of carbon fibers. Using these fibers with a smart design to avoid overuse in composites will help protect the environment, as well as prevent high costs. Cladding panels, one of the two main uses of FRCMs, are generally supported along their length. Higher tensile stresses occur in the length direction. To carry high tensile stresses, a high amount of reinforcement is also required. When stresses are successfully carried along the long span direction, there will be no need for reinforcement in the short span direction. The second use area of FRCM composites is strengthening against earthquake loads. The reinforcements in the direction corresponding to the tensile stress work actively, while in the other direction the reinforcements are subordinate. In the directions where the composite is not subjected to any load or subjected to very low loads, instead of using carbon fibers, the use of e-glass fibers will prevent unnecessary use of carbon fibers. In this way, the aim is to achieve a similar performance by using less carbon fiber or to obtain superior performance by using the same amount of carbon fiber. The performances of composites with high-volume carbon fiber along the beneficial direction: (CL fabrics), carbon along the primary direction and e-glass along the secondary direction, and the composites produced with only carbon: (C fabrics), equal amounts of carbon fiber in both directions, in high-strength matrices were experimentally compared. Ensuring that carbon fibers carry a load close to their capacity is also important for the efficient use of resources. When using commercial carbon fabrics without designing the composite, the loads on the carbon fibers are far below the fiber load-carrying capacity. In previous studies, this has not been emphasized enough. Here, it is necessary to increase the stresses on carbon fibers to use carbon fibers more efficiently. For this reason, it is thought that the use of fabrics produced with yarns containing less fiber and more surface coating could be effective. In this study, in the composites with two different carbon amounts in the same direction in two high-strength cementitious matrices, the stresses on the carbon fibers and on the entire composite section are addressed. In this context, the novelty of the study is, addressing the more efficient use of carbon fabrics in high-strength matrices by employing the carbon fibers in the beneficial direction and changing the amount of coating per unit fiber area.

## 2. Materials and Test Methods

In the experimental study, two different ready dry mix high-strength mortars and two different carbon fabrics were used. The ready mixes used have characteristic strengths of 60 MPa (M60, TICM, Istanbul, Turkey) and 120 MPa (M120, TICM, Istanbul, Turkey). M60 mortar is a thixotropic mortar that can be applied on vertical surfaces, however M120 mortar is a self-leveling mortar. CEM1 52.5 cement (CIMSA, Mersin, Turkey), dry-state polycarboxylate-based superplasticizer, quartz-based aggregate, and mineral additives were used when both of the mortars were produced. The maximum particle size of the quartz-based aggregate used is 1 mm. While F class fly ash is used as a mineral additive in the production of M60 mortar, silica fume is used as the mineral additive in the production of M120 mortar. Mortars are mixed in the laboratory using the amount of water specified by the manufacturer. The mixing speed was 600 rpm. The prepared mortars were cast into 40 mm × 40 mm × 160 mm molds and compressive strengths were obtained after 28 days.

**Figure 1:**
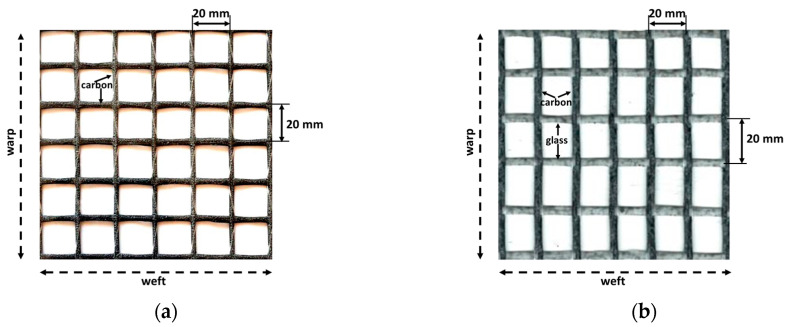
Fabrics used in the production of FRCM composites: (**a**) C fabric; (**b**) CL fabric.

When designing carbon fabrics, the aim is to reveal the effects of the carbon fibers on the beneficial properties of FRCM composites. The tensile strength of the carbon fiber was 3600 MPa according to the producer’s declaration. Two different carbon fabrics were designed for targeted analysis. For the first of these, fabrics with a carbon fiber area of 44 mm^2^/m were designed and produced in the warp and weft directions. It contains equal amounts of carbon fiber in both directions. These fabrics will be abbreviated as (C) in the next part of the paper. For the second type of carbon fabric, all of carbon fibers were assigned in the warp direction. In the weft direction, e-glass glass fibers were used for the assembly of carbon fibers. The amount of carbon fiber in the warp direction in this fabric was 105 mm^2^/m. These fabrics will be abbreviated as (CL) in the next part of the paper. Initially, CL fabric was aimed to use twice as much fiber as C fabric in the warp direction. However, due to the fact that the production technique used on production lines does not allow the use of this ratio, using exactly twice the amount of carbon fiber as the C fabric in the warp direction was not possible. The amount was considered to be 20% more than twice the amount of carbon fiber as the C fabric in the warp direction. Both fabrics are coated with styrene butadiene rubber (SBR) for ease of handling and application. Both fabrics used in the production of the composites are shown in Figure 1.

**Figure 2:**
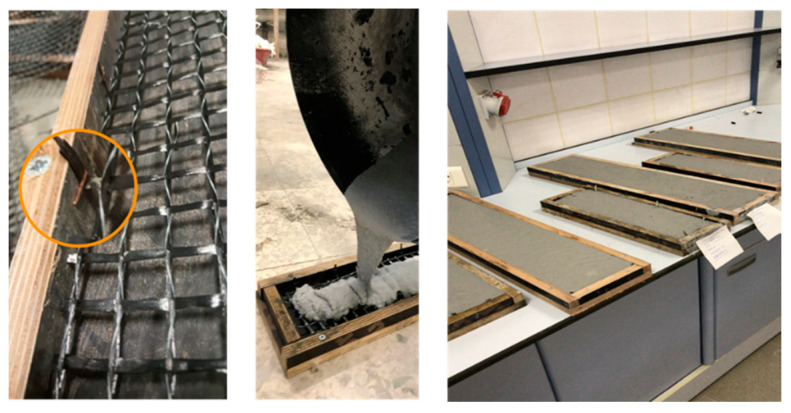
Production stages of composites produced using the M120 matrix.

**Figure 3:**
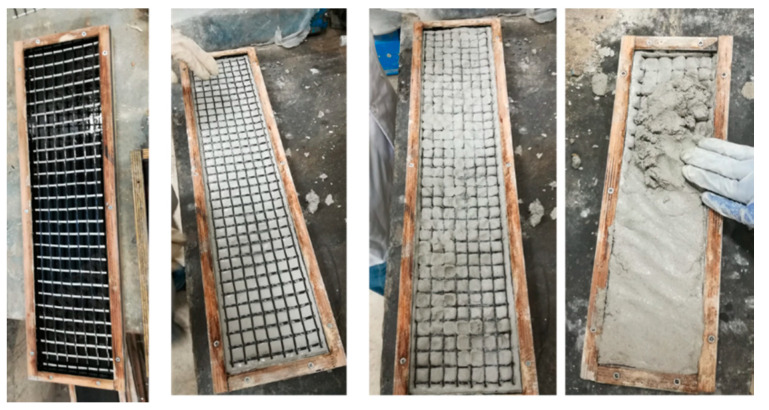
Production stages of composites produced using the M60 matrix.

Plywood molds were prepared according to sample sizes for the production of composite samples. The sample sizes were chosen as 600 mm × 130 mm × 20 mm for tensile test samples and 300 mm × 100 mm × 20 mm for flexural test samples. Since the M120 matrix is self-leveling, the reinforcements were fixed by stapling the mold edges. Then, the self-leveling M120 mortar was poured into the mold and the production of the composite was completed. The production stages of composites produced with M120 matrix are shown in Figure 2. However, since the M60 matrix has a thixotropic structure, no fixation of reinforcements was performed for composites using M60 mortar. For these composites, the first layer of the mortar was poured into the mold. Later, the reinforcements were placed on the mortar. Finally, the second layer of mortar was poured and the production of the composite was completed. The production stages of the composites produced using the M60 matrix are shown in Figure 3.

While creating sample codes, the matrix and carbon fabric codes were used. The first notation in the sample code indicates the matrix, while the second notation represents the carbon fabric type. For example, M120CL refers to the composite produced with a mortar, with a characteristic strength of 120 MPa and containing the fabric in which carbon (CL) is used in one direction.

**Figure 4:**
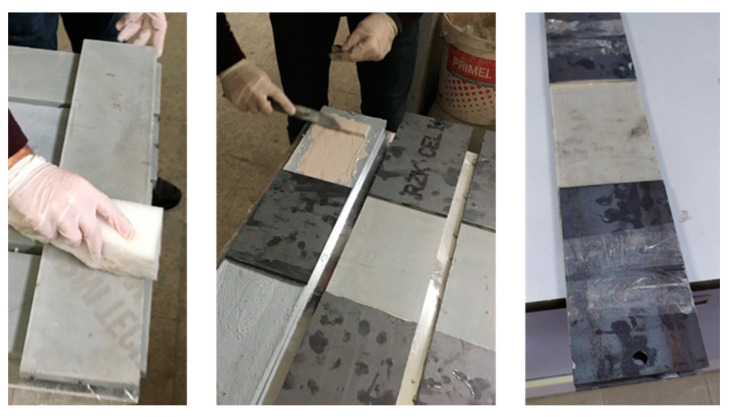
Bonding the steel apparatus to the sample for the tensile tests.

Five samples were produced for each series of experiments. The composites produced were removed from the molds on the day after production and isolated from the external environment using polyethylene stretch film for 26 days until the day of the experiment. The samples prepared for the tensile and flexural tests were then stored in the laboratory until the day of the experiment. For the tensile test, the steel apparatus was glued to the samples as described in the ICC-ES C434 document [53]. The application of the steel apparatus to the tensile samples is shown in Figure 4. No additional apparatus was glued on the specimens for flexural tests.

**Figure 5:**
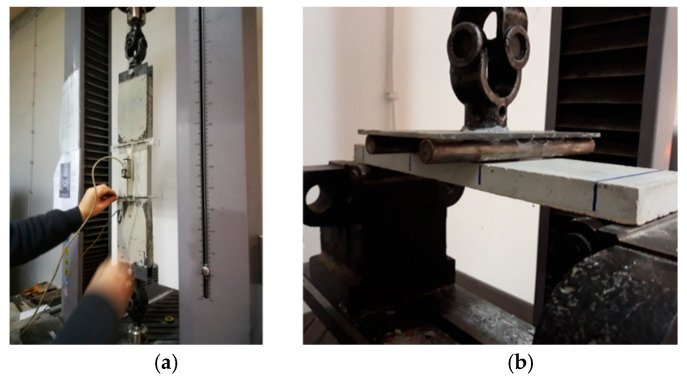
Tensile and flexure test setups: (**a**) tensile test; (**b**) flexure test.

The MTS Criterion C43 504E (MTS Company, 14000 Technology Drive, Eden Prairie, MN, USA) universal deformation-controlled servo test system was used for flexural and tensile tests. The capacity of the test system is 50 kN. The laboratory temperature was measured as 18 ± 2 °C during the tests. Flexural tests were carried out as a four-point bending test. In the flexural tests, the support span length was chosen as 300 mm. During the flexural tests, the strain rate was chosen as 0.6 mm./s. After the metal plates were glued onto the samples in the tensile tests, the metal plates were attached to the steel connectors, which were movable in both directions at both ends. The purpose of applying these steel connectors is not to create any eccentricity during the tensile test. Then, the frame on which deformations were measured was placed on the sample. The length of the frame in which the extensions were measured was 120 mm. Deformations were obtained using linear variable displacement transducers (LVDT) from two points in the middle axis of the sample, at the front and back. The average of the extensions obtained from both LVDTs was taken as the extension of the sample. Tensile tests were carried out with deformation control. Elongation was controlled from the crosshead movement. During the pulling experiments, the crosshead velocity was programmed as 0.8 N/s. The test systems used in the tensile and flexure tests are given in Figure 5a,b, respectively.

## 3. Test Results and Discussion

### 3.1. Compressive Strength Tests for the Mortars

After the samples taken from the mortars were cured in lime-saturated water at 23 ± 2 °C for 28 days, compressive strength tests were applied. As a result of the compressive strength tests, the average strengths of the samples taken from M60 and M120 mortars were obtained as 76 MPa and 132 MPa, respectively.

### 3.2. Tensile and Flexural Test Results of Composites Produced with M60 Matrix

**Figure 6:**
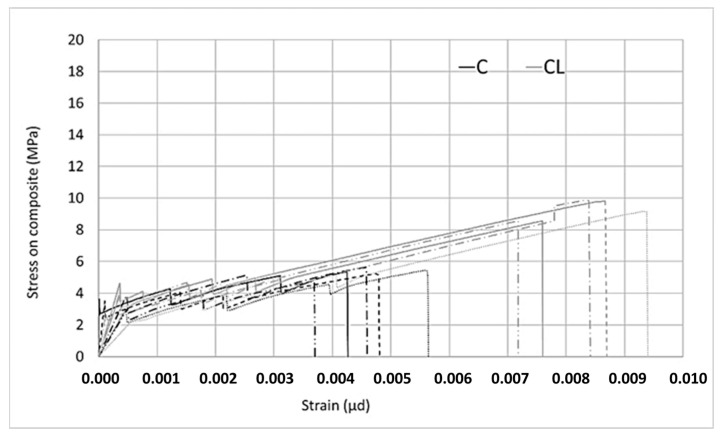
Stress on the composite–strain relationship of the M60 composite.

**Figure 7:**
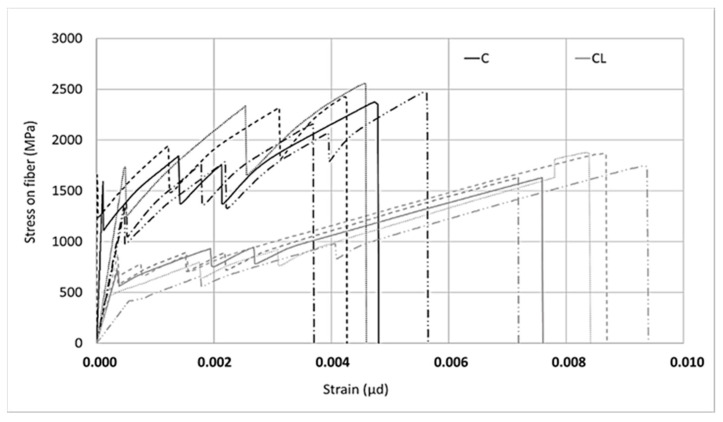
Stress on the fiber–strain relationship of the M60 composite.

Two different types of composites were produced with the M60 matrix. The first composite was obtained by using the M60 matrix and C fabric, which uses carbon fiber in both directions. This fabric uses 44 mm^2^/m carbon fiber in the warp and weft directions. For the second type of composite produced with the M60 matrix, the M60CL composite includes fabric (CL) containing 105 mm^2^/m carbon fiber along the warp direction and e-glass fiber along the weft direction. The graphics obtained from the data for the tensile tests are shown in Figure 6 and Figure 7. Two different graphics were obtained by processing the results of tensile experiments. In both of these graphics, the horizontal axis shows the strain value. However, the stresses on the vertical axes are different. In the graph shown in Figure 6, the vertical axis represents the stress on the composite. The stress on the composite is obtained by dividing the loads obtained in the tensile test by the cross-sectional area of the composite. This value is used in designing cladding applications. However, in the graph shown in Figure 7, on the vertical axis the stress value obtained by dividing the load obtained during the experiment into the fiber area in the direction of the load is presented. In strengthening applications, the load-bearing capacity of the fiber after the section cracks is taken into consideration. At this stage, the design is made considering the stresses on the fiber. After the composite cracks, the fibers become dominant and the stresses on the fibers are effective. For these two approaches the stresses are calculated differently.

According to Figure 6, it is obvious that the relationship obtained is linear up until a certain bend-over point. Beyond this bend-over point, it continues to be linear again, but the slope of the linear relationship between the load and elongation is lower. In the first part of the relationship, the matrix dominates the behavior of the composite, the first crack occurs at the bend-over point, and the fibers dominate the behavior in the second part. When the tensile behaviors of composites produced with C and CL fabrics are examined, the strain hardening part is longer for the CL fabric composite, which indicates a higher load-carrying capacity and higher strain rate.

In both Figure 6 and Figure 7, in the stress–strain curves for the composites in the first region, some differences occur in each series. For example, if the slopes in the first region of the composite produced with C bidirectional carbon are examined, three of the five experiments agree and two of them show steeper slopes. However, one of the composites produced with unidirectional CL fabric shows a lower slope. These differences are not seen in the ultra-high-strength matrix composites. Considering that the ultra-high-strength matrix is a self-leveling mortar, it is thought that these differences in the composites produced with M60 mortar may be caused by possible placement problems.

In Figure 7, for the composite produced with fabric C, in the second part, especially where the fibers are effective, higher stresses were obtained compared to the stresses on the CL fabric. When using less fiber and a larger interface area with the matrix, higher stresses can be transferred to the fibers. As the ratio of the interface layer to the carbon fiber surface per unit increases, the performance obtained from the carbon fiber increases. As the surface area of the coating layer increases, which is the interface in this method, considering that the layer’s adhesion strength to the fibers or matrix is constant, the forces that the interface layer can transfer to the fibers increase. For the behavior in Figure 7, it is clear that there are fewer fibers in the loading direction, a higher–fiber SBR interface, and a higher SBR coating–cement mortar interface per unit area of carbon fiber for the C-fabric-reinforced composite. Thanks to the higher coating interface area, the interface encounters lower stresses against the forces coming onto it and reaches its capacity later. For this reason, the carbon in the loading direction can carry higher tension stresses in fabric C. Since carbon fibers obtained from the same source are used in both fabrics, the effect of the interface performance is clear–having a larger interface area helps to carry more load along the loading direction.

**Figure 8:**
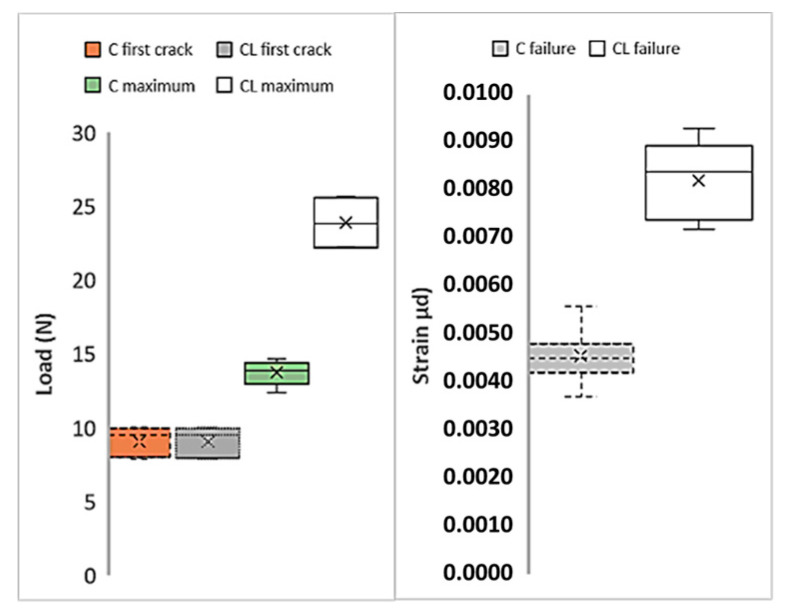
Variations of the critical points of the tensile test results of the M60 matrix composites.

Figure 8 shows the first cracks, maximum loads, and strain values of M60 composites produced with C and CL fabrics. Here, the load values obtained in the tensile test are used directly. In the figure, the average values are given together with the distribution of the results from the five samples. Cross marks indicate the average value of each series. In Figure 8, the first crack loads show lower values for both composites compared to maximum loads. The initial crack values that occur at the end of the region where the matrix is dominant [54] are very close to each other for both C and CL composites. Likewise, the distribution of the values is similar. When the maximum loads are compared, the maximum load of the composite produced with the CL fabric is approximately twice the maximum load of the composite produced with C. This increase in the maximum value can be considered as the effect of the high fiber amount in the CL. Looking at the strain values at the moment of failure, it is understood that the composite produced with CL fabric shows higher strain values than the composite produced with C fabric. The strain values of the composite produced with CL fabric are approximately twice the strain values of the composite produced with C fabric.

**Figure 9:**
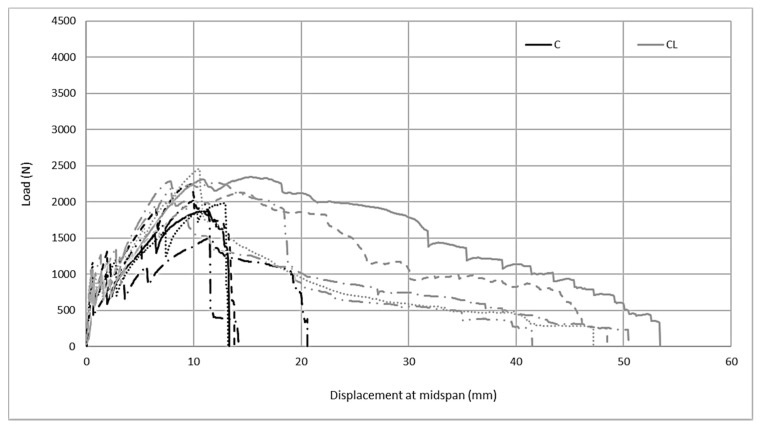
Load midspan displacement relations of the M60 carbon composites at the flexure point.

The examination of the flexural behavior of composites produced with the M60 matrix in Figure 9 shows that there is a linear relationship between the load up to the bend-over point and displacement at the midspan. After this point, the first crack occurs and the multiple crack mechanism initiates bending behavior. In C and CL carbon fabric composites, strain hardening behavior is observed with the multiple crack mechanism in the second part following the bend-over point. As a result of the strain hardening behavior, composites reach their maximum bending capacity. However, the behavior of the composites produced with two fabrics under bending loads differs after the maximum load point. In composites produced with C fabric containing carbon fiber in two directions, after reaching the maximum load, the load suddenly drops to zero. This limits the composite’s energy absorption capacity. However, when the bending test results of the fabrics containing twice the amount of carbon in one direction are examined, strain softening behavior occurs after following the strain hardening behavior and the load gradually decreases until the sample completely fails.

**Figure 10:**
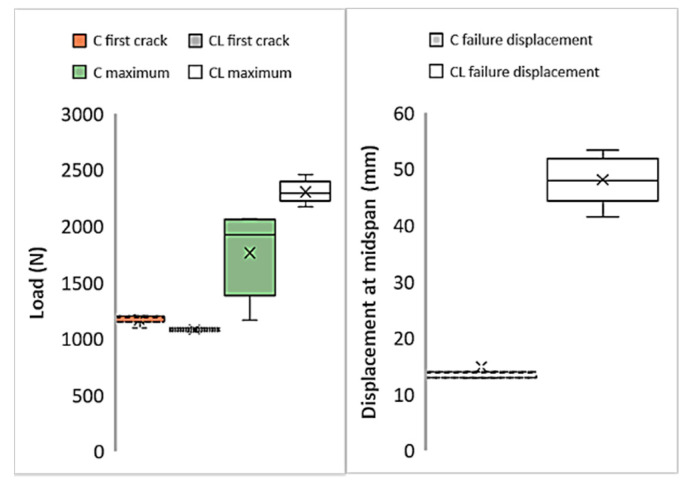
Critical points of the M60 composite’s flexural test results.

Figure 10 shows the first cracks, maximum loads, and failure displacement values in the graphics obtained as a result of flexural tests of composites produced with M60 mortar. Figure 10 also shows the distribution of the test results from 5 samples. Although the first crack values of the composites produced with C fabric are insignificantly higher than composites produced with CL fabric, the load values obtained can be regarded at the same level. The maximum loads carried by the CL fabric composite are considerably higher than the loads produced by the C fabric. However, the high distribution of the maximum load values of composites produced with C fabric is also noteworthy. According to the midpoint displacement values at failure, the midpoint deformation amounts of the composite produced with CL at the time of failure reach three times the deformation amounts of the C composite. This result obtained in the bending test confirms the other results of the tensile test. The composites produced with the CL fabric cause a higher rate of failure deformation. Accordingly, it can clearly be seen that composites produced with CL show more ductile behavior.

### 3.3. Tensile and Flexural Test Results for Composites Produced with the M120 Matrix

**Figure 11:**
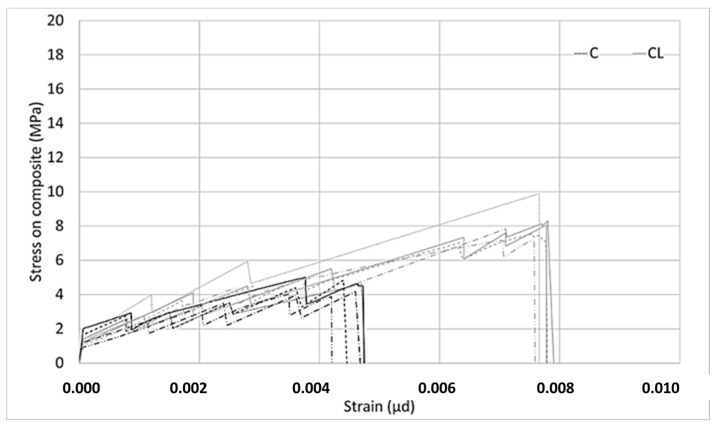
Stress on the composite–strain relationship of the M120 composite.

**Figure 12:**
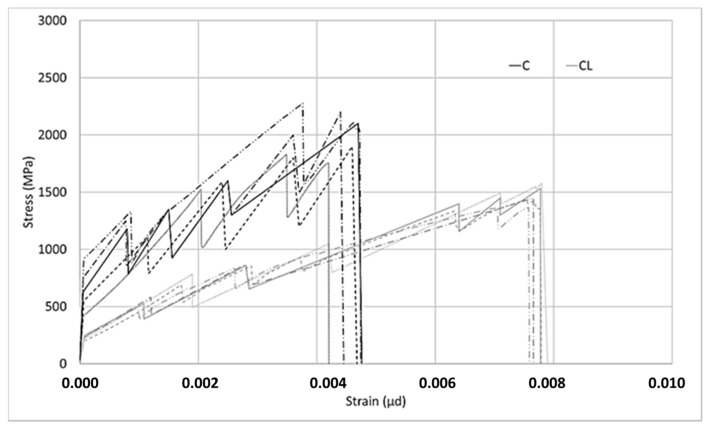
Stress on the fiber–strain relationship of the M60 composite.

The tensile test results of the FRCM composites produced with M120 mortar are presented in Figure 11 and Figure 12. The charts provide graphs of FRCM composites obtained using ultra-high-strength mortar, C, and CL fabrics. Using the tensile test results of composites produced with M120 mortar, two different graphs were obtained. Figure 11 shows the relationships between stresses on the composites and strains. In the composites produced with both fabrics, the stresses on the composites are very close to each other until the first cracks occur. In this part, the slopes between the stress and strain in the composites produced with both fabrics are very close to each other. Since the matrix is dominant in this part, the effects of the fibers on the slope of the line are not seen. After the first cracks have occurred, the stress–strain relationship continues linearly with a lower slope. Strain hardening behavior continues until the maximum stress value in the composite is produced with both fabrics. After the maximum load, a decrease to zero is achieved. In the second part, multiple crack behavior is observed in both composites until it reaches the maximum point. Cracks are reflected in the graphs as instantaneous drops in stress.

In Figure 12, the relationship between the stress on the fiber and the strain is given. According to the graph given in Figure 12, both the M120C and M120CL composites initially have a high slope. When the initial part of the graph is over, the first cracks in the samples are observed. Then, a number of microcracks form until failure in both samples. Following the formation of microcracks, the cracks widen and the experiments end with the growth of one of the multiple cracks formed at the end of the experiment and the failure of the carbon fibers. The slopes in the first part are very close to each other for both composites. This supports the thought that the behavior of the composites in this first region is governed by the matrix. However, after the first cracks occur, the slopes shown by the stress–strain curves of both composites differ from each other. The stress on the fibers at failure is higher for the C composite than for the CL composite. Cracks are reflected in the graphs as instantaneous drops in stress during the second stage.

**Figure 13:**
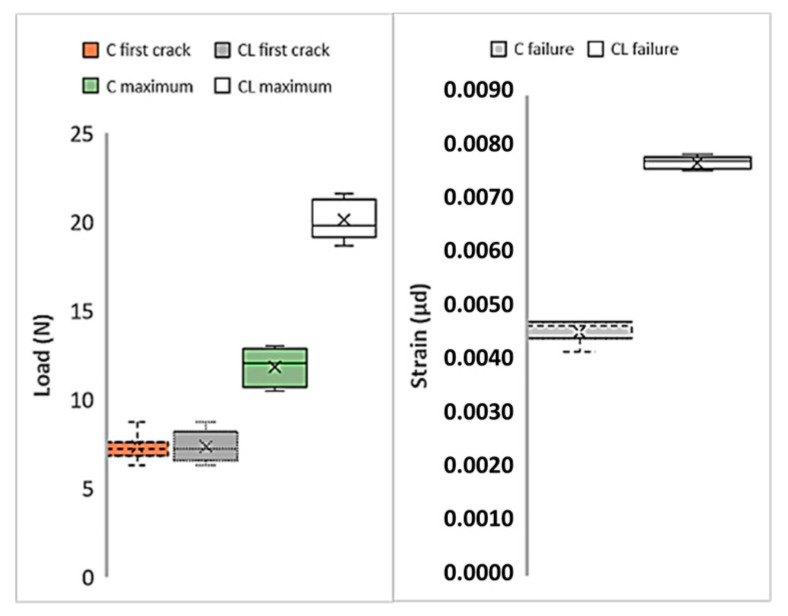
Variations of the critical points of the tensile tests for M120 matrix composites.

For the composites produced with ultra-high-strength M120 mortar, critical points in the tensile test results are shown in Figure 13. When the first crack loads in the C and CL composites are compared, it is clear that no difference can be seen between the average values. The difference between the initial crack loads of the M120C and M120 CL composites is due to the variation of the results obtained from the test samples. While the deviation of the results obtained from the tensile test of the C composite is lower, the distribution of the composite samples produced with the CL sample is higher. According to the comparison of the maximum loads of the composites, the maximum load value of the composite produced with CL fabric is higher than the maximum load value of the composite produced with C fabric. However, the maximum load of the composite produced with CL fabric did not reach double the values of the composites produced with the M120 matrix and C fabric. For the M60 matrix, the maximum load of M60CL was twice as high as M60C. Although the fibers are more effective in the second part of the curve, the stress on the composite remains lower than double that of C, which again is attributed to the performance of the coating in the interface. The CL fabric has a lower coating surface per unit area of fiber along the loading direction. The low surface area causes higher stresses on the coating, which results the coating to fail. This is the result of the tensile performance not reaching twice that of C, although more than twice the amount of fiber is used. If the maximum strain values of the composites produced with C and CL fabrics are compared, it can clearly be seen that the strain values of the composites produced with the CL fabric at the time of failure are higher than those of the composite produced with the C fabric. This high deformation capacity is related to the high load-bearing capacity of the composite produced with the CL fabric.

**Figure 14:**
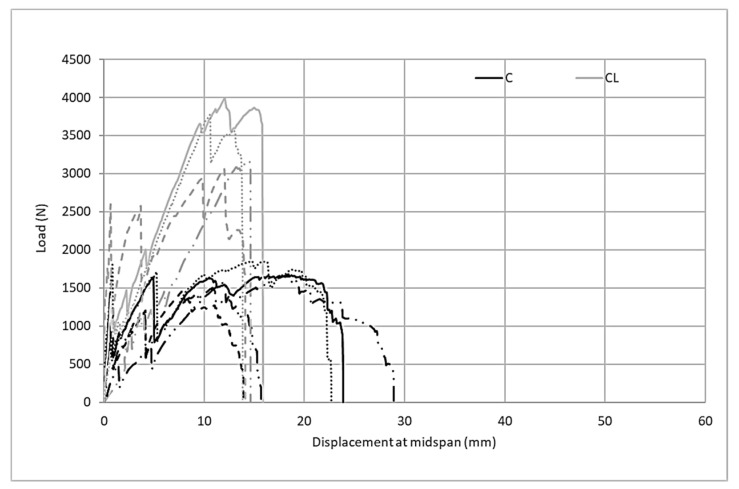
Load midspan displacement relations of M120 carbon composites at the flexure points.

Figure 14 shows the load midspan displacements for the flexure test. The composite samples produced with CL reinforcement have a flexural performance that is approximately 2 times higher than composite samples produced with C reinforcement. The load-bearing capacity under the effects of bending increases more than the amount of fiber. The reason for this performance increase that occurs with the increase of the fiber ratio can be attributed to the high adhesion performance of the SBR coating in the fiber–matrix interface with high-performance mortar. In this way, carbon fibers can be employed as intended against tensile loads. Unlike the M60 matrix, the displacement quantities shown by the composites under bending loads do not differ significantly. Composites produced with CL fabric show a sudden load drop after reaching the maximum load reached after strain hardening. In composites produced with C fabric, the obtained strain softening behavior following the strain hardening behavior is opposite to that of the composite performance produced with the M60 mortar. However, the difference in composites produced with the high-strength matrix is that when the C fabric is used, the strain softening period is shorter than for the composite produced with the M60CL composite; that is, the deformation in this part is lower. The lower deformation can be attributed to the high adhesion performance between the high-performance grout matrix and the carbon surface coating.

**Figure 15:**
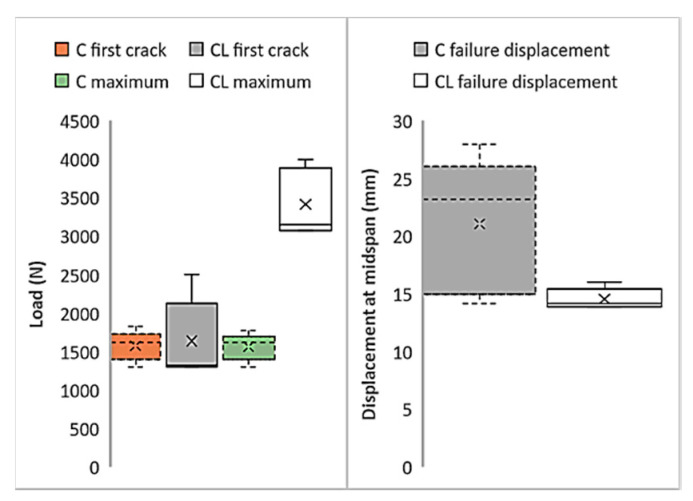
Critical points of the M60 composite’s flexural test results.

Figure 15 shows the critical points obtained from the bending tests of the composites obtained using the ultra-high-strength M120 matrix and both types of carbon fabrics. According to the initial crack loads, it is understood that the average first crack loads for composites produced with C and CL fabrics are very close to each other. However, the variability of the five tested samples of the M120CL composite is higher than the distribution of the flexural test results of the samples of the M120C composite. The maximum load of the composite produced with the C fabric in the flexural test is in the same order as the first crack load. The maximum load of the composite produced with the CL fabric is more than twice the maximum load of the composite produced with C. The M120C composites show a great distribution in the test results. The average failure deformation value for the composites produced with C fabric is higher than the average deformation at the moment of failure for the M120CL composite. This behavior can be attributed to the high shear rates and high deformation caused by the larger interface area.

### 3.4. Microstructural Investigation

**Figure 16:**
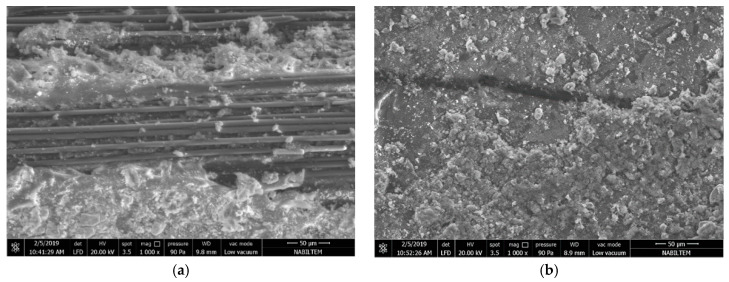
Microstructure view of the M60C composite fibers: (**a**) with peel off; (**b**) without peel off

In Figure 16a, the microstructure of the carbon fibers is shown for the failed M60C tensile test specimens. It can be seen that the SBR coating on the fibers peeled off, together with the cement matrix. This indicates that the adherence between the M60 matrix and the SBR coating is sufficient for transferring the loads to the carbon fiber, however the adherence between the carbon fiber and the coating failed partly at the end of the experiment. This indicates that the optimum strength in the fiber–matrix transition phase was achieved and the increases in matrix strength after this point did not have an impact on performance because there is no significant increase in the performance of composites produced with the M120 matrix. To further improve the performance, the carbon fiber coating needs further improvement.

Figure 16b shows the M60C composite, SBR coating without peel-off, and the cementitious matrix residues on it. Since the adherence between the carbon fiber and the SBR coating is higher in this part of the fabric than the tensile strength of the cement matrix, some residue remains on the coating, causing the cementitious matrix to fail. The failure mode here varies according to the possible defects in the fabric–coating interfaces of the industrially produced fabrics. Peel-off occurs in the region where the defects occur. In the regions where there are no defects, some matrix material remains on the fabric and the stripping of the matrix occurs. It is clear here that the coating thickness of the carbon fabric is not equal at all points. The SBR coating is thin in parts of the peel-off zones. The shear and tensile resistance of the SBR coating, which resists peel-off forces, decreases in thin sections of the coating.

**Figure 17:**
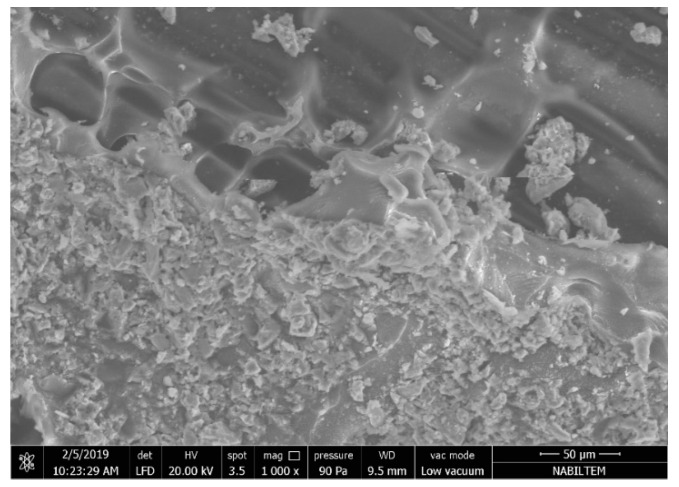
Fabric–matrix interface in the composite produced with M120 mortar.

In Figure 17, the fabric matrix interface of the composite produced with the M120 matrix is presented. The figure shows the SBR coating (the thickness of which is not homogeneous) on the carbon fabric. When the thickness of the fabric coating become non-homogenous, the stresses on this coating and its interface with the matrix also vary. This situation causes different failure modes, even on a small section. Figure 17 is a good example of this. It can clearly be seen that in the 300 × 250 micrometer area, assuming that the load during the experiment is equal, different failures are formed on the interface even, within a very small zone.

**Figure 18:**
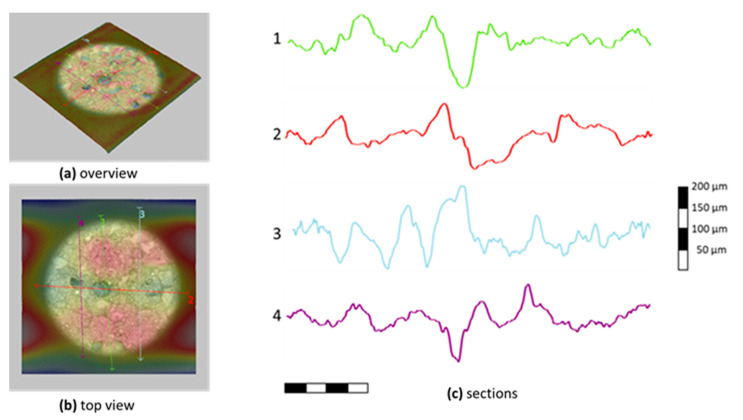
Microtopography of carbon fabric after failure in M60 composites.

**Figure 19:**
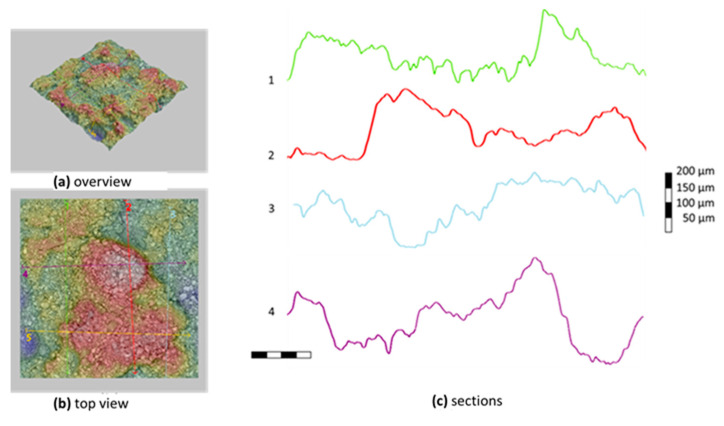
Microtopography of carbon fabric after failure in M120 composites.

In Figure 18 and Figure 19, the microtopographies and sections of carbon fabric surfaces after the tests are presented for M60 and M120 matrix composites, respectively. These sections are taken under a phenom scanning electron microscope. The sections given in the figures were selected to represent the majority of the samples. In this part measurements from the peeled-off parts were avoided. Here, the topographic section of the mortar residue on the coating was examined in an area measuring approximately 1 mm × 1mm and the adhesion capacities of the matrices on the fabric were evaluated. In the M60 matrix mortar composite, the mortar residue height of 100–150 micrometers mostly remained on the fabric and displayed a homogeneous bonding. In the micro topographies of composites produced with the M120 matrix, the maximum mortar residue height in the studied area was around 250–400 micrometers. This shows that the high-strength mortar has better bonding performance with the carbon fabric coating.

## 4. Conclusions

The more efficient usability of carbon fabrics in high-strength cementitious matrices by employing the carbon fibers in the beneficial direction and changing the amount of coating per unit fiber area was investigated experimentally. The following conclusions can be drawn according to the results obtained from the experimental study, within the limits of the materials used:

- In the experiments on the composite, regardless of the amount of fiber in the direction of loading, the parts of the graphs until the first crack occurs depend on the matrix properties and are managed by the matrix. After the first cracks had occurred, the multiple cracking mechanism was activated in all samples in tensile and flexural tests and strain hardening behavior was observed. In tensile tests, the load suddenly dropped to zero after the maximum load was reached in all of the samples, and then failure occurred. In flexural tests, while samples produced with the CL fabric and M60 composites showed strain softening behavior, composites produced with M120 mortar showed similar strain softening behavior when using the C fabric. The strain softening behavior affects the ductility of the sample in flexural tests. If carbon fibers are used in a beneficial direction, the mechanical energy absorbed in the stage after the maximum load is reached is higher;
- The first crack loads were not significantly different when different fabrics were used for flexure and tensile tests. The maximum loads increased when CL fabric was used. With the increase of fiber area in the direction of the load, the load order carried along this direction increases. However, this increase does not have to be proportional to the fiber area used in the direction of the load. Improvement of the matrix properties over 60 MPa has no significant effect on composite mechanical properties;
- Regarding the performance of high-strength cementitious mortar and carbon fiber composites, the interface of the coatings with the carbon fiber or cementitious matrix affects the performance. The fibers, fiber–matrix interface, coating material, and matrix properties should be optimized to give best performance for the composite. Optimization can be done by modeling the peel-off strengths of the coating on the fibers. It should be noted that the defects in the interface and elements that disrupt the homogeneity significantly affect composite performance;
- When the residual amounts of high-strength cementitious matrix on the SBR-coated carbon fiber fabric were examined, it was clear that the SBR coating provided sufficient adherence for the cementitious matrix. In the microtopography studies, it was found that the high-strength matrix had better adhesion to the coating. However, factors such as the coating type and coating thickness should be taken into consideration regarding the composite performance;
- The thickness of the coating takes advantage of the performance of the fibers and transfers the loads on the coating to the fibers. The parts with low coating thickness were peeled off over the carbon fibers;
- By reducing the amount of carbon fiber along the load-carrying direction, the amount of coating corresponding to the unit area of the carbon fiber is increased. The carbon fibers can be used more efficiently and the loads in the matrix can be transferred to the carbon fibers through the coating–matrix interface, allowing the carbon fibers to carry more loads. Thus, carbon fibers can be used more efficiently by carrying closer loads. It has been demonstrated that the increase in the ratio of the carbon fiber to the cement matrix interface per unit area of carbon fiber is effective in increasing the load transferred to carbon fibers. Using more carbon fibers in one direction increases the load-bearing capacity of the composite in terms of tensile strength and bending. However, using a higher yarn numbers with thinner yarn will further increase the tensile stresses on the fiber and the overall load-carrying capacity;
- Further studies could investigate which coatings might be optimized by performing experimental studies on different coating types, as well as which carbon fiber thicknesses should be used.
